# *Leishmania donovani* Infection Causes Distinct Epigenetic DNA Methylation Changes in Host Macrophages

**DOI:** 10.1371/journal.ppat.1004419

**Published:** 2014-10-09

**Authors:** Alexandra K. Marr, Julia L. MacIsaac, Ruiwei Jiang, Adriana M. Airo, Michael S. Kobor, W. Robert McMaster

**Affiliations:** 1 Immunity and Infection Research Centre, Vancouver Coastal Health Research Institute, Department of Medical Genetics, University of British Columbia, Vancouver, Canada; 2 Centre for Molecular Medicine and Therapeutics, Child and Family Research Institute, Department of Medical Genetics, University of British Columbia, Vancouver, Canada; 3 Human Early Learning Partnership, School of Population and Public Health, Department of Medical Genetics, University of British Columbia, Vancouver, Canada; University of Dundee, United Kingdom

## Abstract

Infection of macrophages by the intracellular protozoan *Leishmania* leads to down-regulation of a number of macrophage innate host defense mechanisms, thereby allowing parasite survival and replication. The underlying molecular mechanisms involved remain largely unknown. In this study, we assessed epigenetic changes in macrophage DNA methylation in response to infection with *L. donovani* as a possible mechanism for *Leishmania* driven deactivation of host defense. We quantified and detected genome-wide changes of cytosine methylation status in the macrophage genome resulting from *L. donovani* infection. A high confidence set of 443 CpG sites was identified with changes in methylation that correlated with live *L. donovani* infection. These epigenetic changes affected genes that play a critical role in host defense such as the JAK/STAT signaling pathway and the MAPK signaling pathway. These results provide strong support for a new paradigm in host-pathogen responses, where upon infection the pathogen induces epigenetic changes in the host cell genome resulting in downregulation of innate immunity thereby enabling pathogen survival and replication. We therefore propose a model whereby *Leishmania* induced epigenetic changes result in permanent down regulation of host defense mechanisms to protect intracellular replication and survival of parasitic cells.

## Author Summary

The *L. donovani* parasite causes visceral leishmaniasis, a tropical, neglected disease with an estimated number of 500,000 cases worldwide. Current drug treatments have toxic side effects, lead to drug resistance, and an effective vaccine is not available. The parasite has a complex life cycle residing within different host environments including the gut of a sand fly and immune cells of the mammalian host. Alteration of host cell gene expression including signaling pathways has been shown to be a major strategy to evade host cell immune response and thus enables the *Leishmania* parasite to survive, replicate and persist in its host cells. Recently it was demonstrated that intracellular pathogens such as viruses and bacteria are able to manipulate epigenetic processes, thereby perhaps facilitating their intracellular survival. Using an unbiased genome-wide DNA methylation approach, we demonstrate here that an intracellular parasite can alter host cell DNA methylation patterns resulting in altered gene expression possibly to establish disease. Thus DNA methylation changes in host cells upon infection might be a common strategy among intracellular pathogens for their uncontrolled replication and dissemination.

## Introduction

*Leishmania* parasites have a complex life cycle usually alternating between an insect vector and a vertebrate host, or between vertebrate hosts. The parasite is spread to humans through sandflies of the genus *Phlebotomus* or *Lutzomyia* during a blood meal [1]. Within the mammalian host, *Leishmania* infect macrophages, cells that play a critical role in regulation of immune system and in host defense [2]. Pivotal to cellular immune responses, macrophages function as antigen processing and presenting cells and produce a variety of cytokines that have pleiotropic effects within the host. *Leishmania* have evolved to evade the defense mechanism of these cells through inhibition of macrophage activation that enables pathogen replication and survival [3]–[6]. For example, essential macrophage activation signaling molecules and pathways such as PKC, JAK/STAT, MAPK, NF-kB as well as the transcription factor AP-1 are deactivated following infection with *Leishmania* [7]. In addition, molecules such as SHP-1 are activated during *Leishmania* infection causing SHP-1 mediated JAK2 inactivation in macrophages [7]. Thus *Leishmania* evolved several strategies to inhibit macrophage activation, the ability to present antigens on their surface as well as to interfere the communication of macrophages with cells from the adaptive immune system [7].

Molecular mechanisms of cell programming often involve epigenetic changes by chromatin remodeling, histone modifications, and/or DNA methylation leading to regulation of cellular gene expression for normal development and establishing and maintaining cellular differentiation [8]. DNA methylation, the addition of a methyl group to the 5′ cytosine primarily in the context of CpG dinucleotides, is arguably the most commonly studied epigenetic mark. While shaping the cellular DNA methylation patterns is in large parts a developmental- and tissue-specific dynamic process [9], recent work suggest that it can be affected also by a broad variety of environmental factors [10]. CpG dinucleotides are not randomly distributed across the genome; rather, they are enriched in relatively infrequent distinct stretches of DNA termed “CpG islands” [11], over half of which are located in known promoter regions of genes [12]. These regions can be further classified into high, intermediate, intermediate shore, and low categories, based on their CpG density [12]. Generally, high levels of DNA methylation in promoter regions are associated with decreased gene expression and vice versa, but this relationship is not always straightforward [13]. Changes in DNA methylation patterns that occur mainly in proximate promoter regions, but also in gene body regions, can result in aberrant gene transcription of associated genes [13]–[15].

The field of microbe-induced epigenetic changes in host cells is just starting to be explore [16]–[18]. Recently, microbe-induced epigenetic changes in host cells emerged as a mechanism whereby intracellular pathogens such as viruses and bacteria manipulate host processes to favour their intracellular survival [16], [17], [19]. Alterations in macrophage DNA methylation in response to intracellular protozoan pathogens remains largely unknown and permanent inhibition of innate immune response could be explained by changes to the host cell epigenome. In this study we set out to test the intriguing hypothesis that *L. donovani* induces epigenetic changes in DNA methylation of the human macrophage genome. Using unbiased DNA methylation array technology a set of CpG sites was identified with changes in methylation that correlated with live *L. donovani* infection. These loci occurred in regions with distinct CpG densities and affected signaling pathways associated with host defense. Collectively, this work suggests that *L. donovani* causes specific effects on the epigenome of the macrophage host, which might enable better survival.

## Results

### Genome-wide analysis to quantify methylation of single CpG sites in the human macrophage genome

**Figure 1:**
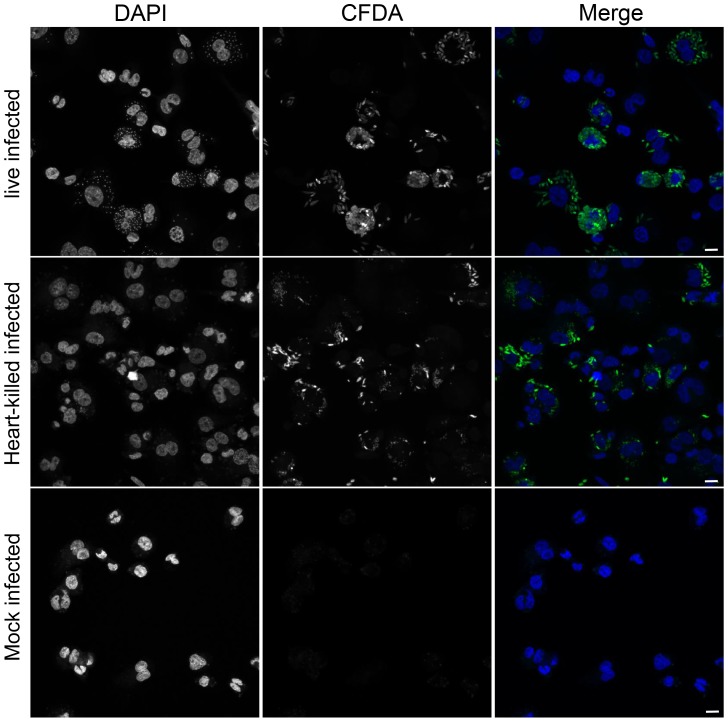
Heat-killed *L. donovani* are phagocytosed by THP1 cells. THP1 cells that had been infected for 24 hr with CFDA pre-stained (green) live or heat-killed *L. donovani*. Heat-killing was performed after loading live *L. donovani* promastigotes with CFDA (see material and method section). DAPI (blue) was added to stain the nucleus and kinetoplast. Scale bar represents 25 µm.

To evaluate epigenetic changes in host cells caused by infection with a protozoan parasite, DNA methylation of genomic DNA from human macrophages infected with *L. donovani* was studied. DNA methylation of CpG sites in the genome of host cells was quantified using the Illumina Infinium HumanMethylation450 BeadChip array. This technology allows for the quantitative measurement of DNA methylation at over 480,000 CpG dinucleotides, broadly representing promoter and coding regions of almost all RefSeq genes [20]. To differentiate among changes induced specifically by *Leishmania* infection versus those triggered by phagocytosis, macrophages treated with heat killed *L. donovani* promastigotes, as well as uninfected macrophages were used as controls. Three biological replicates were performed for each experimental condition. The infection rates of the three independent experiments were very similar (experiment 1: 81%, experiment 2: 79% and experiment 3: 83%). Overall, the correlations between replicates for the same treatment were slightly higher than those between the treatments (r = 0.998 and r = 0.997 respectively). Using unsupervised clustering, we found that individual samples from specific treatments did not necessarily cluster next to each other (Figure S1). To monitor whether heat-killed *Leishmania* were successfully phagocytosed by the THP1 cells, CFDA pre-stained *Leishmania* (either live or heat-killed) were used to infect THP1 cells and then processed for confocal fluorescence microscopy. Both, live- as well as heat-killed *Leishmania* were phagocytosed by THP1 cells (Figure 1). Collectively, these data suggest that *L.donovani* infection of macrophages did not result in wholesale changes to the host DNA methylome.

### Identification of changes in the DNA methylation pattern in the host cell genome upon infection with *L. donovani*

To more carefully investigate whether infection with *L. donovani* caused DNA methylation changes at specific genes in host epigenome, we performed linear modeling with the R limma package using all possible pairwise comparisons among the three groups of samples (live infected, heat killed treated, and uninfected) [21]. Probes with a p-value of 0.05 or less after Benjamini Hochberg correction for multiple testing were considered significantly differently methylated between the groups. Changes in DNA methylation were expressed as Δ Beta values, defined as the difference between mean DNA methylation of a sample group and mean DNA methylation of control samples (heat killed treated or uninfected) at a particular probe. A detailed description of the analysis is provided in the methods section.

**Figure 2:**
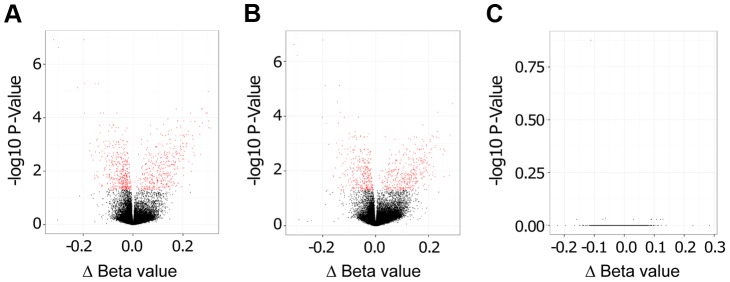
*L. donovani* infected human macrophages showed significant DNA methylation changes compared to control cells. Volcano plot showing differentially methylated sites for the three comparisons. **A**: *L. donovani* live infected versus uninfected macrophages. **B**: *L. donovani* live infected versus heat killed treated macrophages. **C**: *L. donovani* heat killed treated macrophages versus uninfected macrophages. For the comparisons of live infected versus uninfected and versus heat killed treated, there were many CpG sites with differentially methylated CpG probes (red); however no differentially methylated CpG sites were found for the comparison between heat killed treated and uninfected macrophages.

Importantly, as evidenced by Volcano plots that display −log_10_ P-Values versus Δ Beta values, we found a large number of statistically significant changes in CpG methylation (coloured in red in Figure 2) when comparing live promastigote infected versus uninfected macrophages (Figure 2A), and live promastigote infected versus heat killed promastigote treated macrophages (Figure 2B). In contrast, no statistically significant different methylated CpG sites were identified when comparing heat killed treated versus uninfected macrophages, demonstrating that phagocytosis does not alter methylation of macrophage CpG sites (Figure 2C). These data strongly suggested that infection with *L. donovani* indeed resulted in specific changes in the macrophage host DNA methylome.

**Figure 3:**
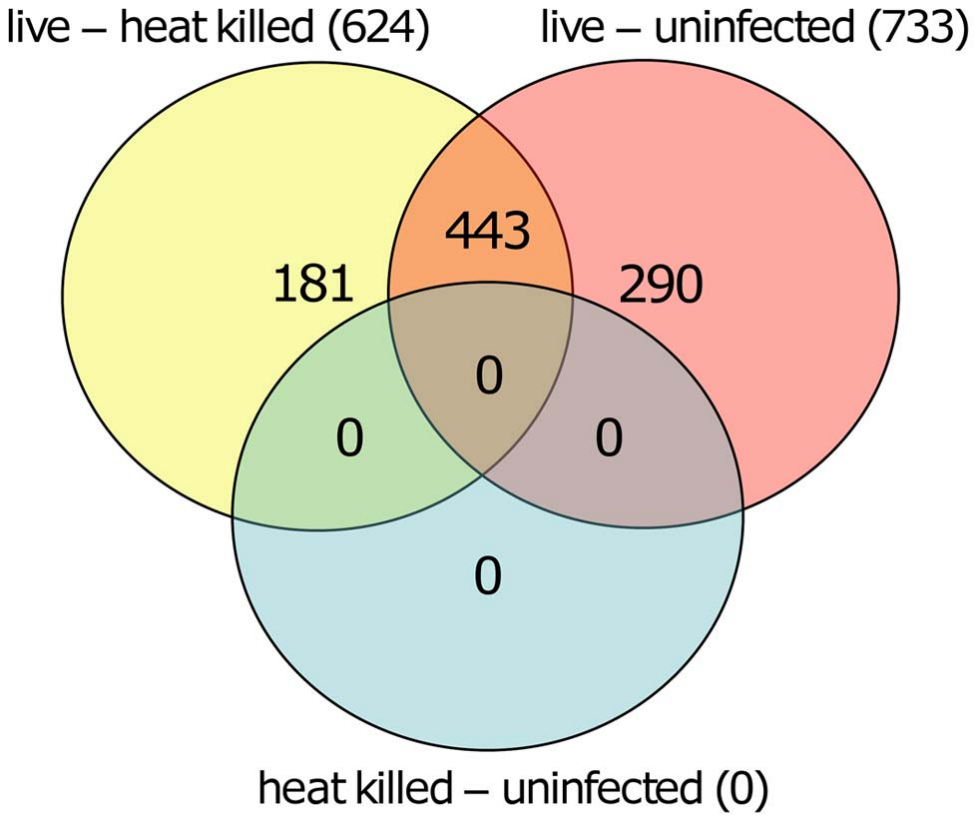
Overlapping CpGs identify high confidence loci for specific effects of *L. donovani* infection. VennDiagram of CpG sites that were significantly different methylated in human macrophages when comparing live promastigote infected versus heat killed treated (live – heat killed), live promastigote infected versus uninfected (live – uninfected) and heat killed versus uninfected (heat killed – uninfected). A total of 624 CpG sites were found significant different methylated between live promastigote infected and heat killed treated macrophage DNA samples whereas 733 CpG sites were found significantly different methylated between live promastigote infected and uninfected macrophage DNA samples. 443 CpG sites were commonly found in both comparisons, thus representing a high confidence set for specific effects of *L. donovani* infection. Comparison of single CpG site methylation status was done using limma. Multiple testing correction was done using the Benjamini-Hochberg method (P values<0.05).

We next quantified and compared the number of statistically significant differentially methylated CpGs between the live infected versus uninfected macrophages and live infected versus heat killed treated macrophages respectively. Given that no significant changes in DNA methylation status of CpG sites were observed in heat killed treated versus uninfected macrophages (Figure 2C), this group was not analyzed further. Using the criteria outlined above, we determined 733 and 624 CpG sites with altered methylation between live infected versus uninfected macrophages and live infected versus heat killed treated macrophages, respectively (Table S1, Table S2, and Figure 3). To derive a high confidence set of CpGs whose methylation was specific for *Leishmania* infection as opposed to being triggered by phagocytosis, we focused on the subset of 443 CpG sites that were significantly different in both live promastigote infected versus heat killed treated macrophage and live promastigote infected versus uninfected macrophage data sets (Table S3).

### Analysis of differentially methylated CpG sites

The 443 CpGs from our overlapping high confidence set all had changes in the same direction when comparing their original conditions, although their absolute magnitude differed for some CpGs between the two (Table S3). 315 of the 443 CpG sites were associated with a gene ID (Table S3). Overall, in the high confidence group there was a slightly larger fraction of CpGs that had decreased methylation compared to increased methylation (51.47% versus 48.53%) (Table S3). Next, we filtered significant loci for absolute change in DNA methylation, as we reasoned that larger differences might be more likely to exert biological effects. For live infected versus heat killed with a 10% delta beta cutoff, 37 sites decreased and 135 increased in DNA methylation, and with a 20% cutoff, 3 sites showed a decrease and 23 an increase (Table 1). For live infected versus uninfected with a 10% delta beta cutoff, 38 sites decreased and 147 increased, and with a 20% cutoff three showed a decrease and 31 an increase (Table 1). The largest differences in absolute magnitude of CpG methylation (i.e. statistically significant when compared between live infected and control macrophages) are listed in Table 2 (25 CpGs that gained methylation and 25 CpGs that lost methylation).

**Table 1:** Quantification of CpG sites with largest difference in methylation following *L. donovani* infection.

Δ Beta changes	live - heat killed	live - uninfected
**Increase in methylation**		
** >5%**	199	200
** >10%**	135	147
** >20%**	23	31
**Decrease in methylation**		
** >5%**	111	116
** >10%**	37	38
** >20%**	3	3

Quantification of CpG sites with largest differences in methylation following *L. donovani* infection. Number of Δ Beta values for the high confidence list with changes >5%, >10%, and >20% subdivided into decreased and increased methylation of live infected versus heat killed treated, and live infected versus uninfected macrophages.

**Table 2:** 50 CpG sites with largest differences in methylation in live infected versus control macrophages.

decreased methylation									
Infected vs Uninfected					Infected vs Heat killed				
	CpG site	Δ Beta Infected vs Uninfected	UCSC RefGene accession	gene name		CpG site	Δ Beta Infected vs Heat killed	UCSC RefGene accession	gene name
**1**	cg14339867	−0.320217	NM_138430	ADPRHL1	**1**	cg14339867	−0.30940373	NM_138430	ADPRHL1
**2**	cg13526469	−0.300438			**2**	cg13526469	−0.29503371		
**3**	cg10786876	−0.221791	NM_001958	EEF1A2	**3**	cg10786876	−0.20131398	NM_001958	EEF1A2
**4**	cg03826759	−0.196666	NM_031898	TEKT3	**4**	cg03826759	−0.1986742	NM_031898	TEKT3
**5**	cg04085039	−0.194923	NM_020825	CRAMP1L	**5**	cg04085039	−0.18669018	NM_020825	CRAMP1L
**6**	cg09901100	−0.176039			**6**	cg25889035	−0.17703301	NM_014914	AGAP1
**7**	cg25889035	−0.172598	NM_014914	AGAP1	**7**	cg09901100	−0.15134023		
**8**	cg06554036	−0.152636			**8**	cg03611151	−0.14770069	NM_001841	CNR2
**9**	cg13286582	−0.151022	NM_006449	CDC42EP3	**9**	cg13286582	−0.14616033	NM_006449	CDC42EP3
**10**	cg03611151	−0.146611	NM_001841	CNR2	**10**	cg06554036	−0.14380969		
**11**	cg06638568	−0.145707	NM_152410	PACRG	**11**	cg02070232	−0.14040662	NM_207437	DNAH10
**12**	cg20094085	−0.144355	NM_032439	PHYHIPL	**12**	cg14987354	−0.13960631		
**13**	cg18845578	−0.143963	NM_006037	HDAC4	**13**	cg26916687	−0.13699842		
**14**	cg14987354	−0.139285			**14**	cg00863716	−0.13687198	NM_205845	AKR1C2
**15**	cg26916687	−0.138835			**15**	cg02951695	−0.129740287	NM_005539	INPP5A
**16**	cg00863716	−0.134696	NM_205845	AKR1C2	**16**	cg18845578	−0.12824658	NM_006037	HDAC4
**17**	cg02070232	−0.133713	NM_207437	DNAH10	**17**	cg06638568	−0.12809977	NM_152410	PACRG
**18**	cg26684363	−0.131870			**18**	cg17401890	−0.12686627	NM_024841	PRR5L
**19**	cg07861603	−0.131572	NM_001103175	CCDC64B	**19**	cg07861603	−0.12683179	NM_001103175	CCDC64B
**20**	cg21922468	−0.126621			**20**	cg20094085	−0.12508938	NM_032439	PHYHIPL
**21**	cg02951695	−0.125837	NM_005539	INPP5A	**21**	cg27650678	−0.124031	NM_022172	PC
**22**	cg24667405	−0.125342	NM_018490	LGR4	**22**	cg19065773	−0.12298969	NM_152309	PIK3AP1
**23**	cg16075139	−0.124218	NM_004356	CD81	**23**	cg21922468	−0.12136641		
**24**	cg27650678	−0.119544	NM_022172	PC	**24**	cg00934882	−0.11875402		
**25**	cg00934882	−0.119431			**25**	cg05838113	−0.11639465	NM_001164490	ADAM8

increased methylation									
Infected vs Uninfected					Infected vs Heat killed				
0	CpG site	Δ Beta Infected vs Uninfected	UCSC RefGene accession	gene name		CpG site	Δ Beta Infected vs Heat killed	UCSC RefGene accession	gene name
**1**	cg26700919	0.204906	NM_181482	C18orf1	**1**	cg09244312	0.192298468		
**2**	cg05242190	0.205884			**2**	cg09477895	0.19832231		
**3**	cg21211645	0.212834	NM_015340	LARS2	**3**	cg21670717	0.202329497		
**4**	cg20300541	0.213960			**4**	cg08035694	0.205312478		
**5**	cg01127300	0.215136			**5**	cg18527651	0.206970955	NM_001570	IRAK2
**6**	cg07062336	0.221825			**6**	cg21211645	0.208866553	NM_015340	LARS2
**7**	cg22328837	0.227626	NM_001015051	RUNX2	**7**	cg25101764	0.210250025		
**8**	cg06844930	0.230710	NM_020337	ANKRD50	**8**	cg26280911	0.211962048	NM_015288	PHF15
**9**	cg18527651	0.232183	NM_001570	IRAK2	**9**	cg00502926	0.212132793	NM_032572	RNASE7
**10**	cg11760395	0.237771			**10**	cg26055899	0.213358921	NM_015196	KIAA0922
**11**	cg02104162	0.238349			**11**	cg12081267	0.217845134	NM_015348	TMEM131
**12**	cg09776041	0.241004	NM_018161	NADSYN1	**12**	cg17692230	0.218587885		
**13**	cg12081267	0.244316	NM_015348	TMEM131	**13**	cg20300541	0.224045513		
**14**	cg21670717	0.247547			**14**	cg02860705	0.225041525		
**15**	cg14014590	0.247902	NM_004560	ROR2	**15**	cg00006090	0.228083255	NM_031293	PMFBP1
**16**	cg00006090	0.266889	NM_031293	PMFBP1	**16**	cg24475517	0.229486555	NM_019044	CCDC93
**17**	cg09324018	0.267813	NM_030797	FAM49A	**17**	cg25598890	0.23255689	NR_031766	TESC
**18**	cg02860705	0.270031			**18**	cg06844930	0.23437168	NM_020337	ANKRD50
**19**	cg26877720	0.276057	NM_031453	FAM107B	**19**	cg11760395	0.245220432		
**20**	cg25598890	0.277422	NR_031766	TESC	**20**	cg05622577	0.255390047		
**21**	cg26280911	0.278838	NM_015288	PHF15	**21**	cg07063068	0.257433144	NM_003485	GPR68
**22**	cg11737318	0.292699			**22**	cg22328837	0.258857685	NM_001015051	RUNX2
**23**	cg07063068	0.303677	NM_003485	GPR68	**23**	cg10814005	0.260887592	NM_003485	GPR68
**24**	cg00722081	0.304068			**24**	cg00722081	0.281186271		
**25**	cg10814005	0.312177	NM_003485	GPR68	**25**	cg26877720	0.289534351	NM_031453	FAM107B

50 selected CpG sites of the high confidence list (Table S3) with largest differences in absolute magnitude of CpG methylation (25 for decreased methylation and 25 for increased methylation) when comparing live infected versus control (uninfected or heat killed treated) macrophage DNA. In: live infected, Un: uninfected, Hk: heat killed treated.

**Figure 4:**
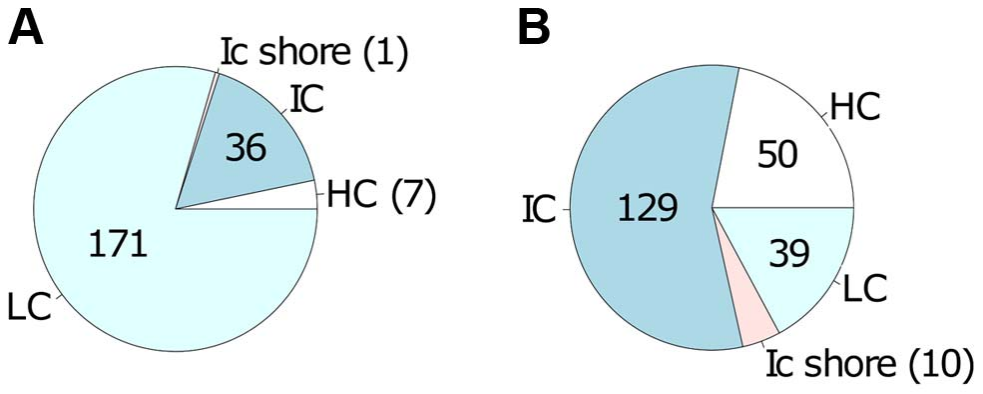
Differentially methylated CpG sites localized mainly in low and intermediate CpG density regions of host cell DNA. Localization of the 443 differentially methylated CpG sites comprising the high confidence set **A**: localization of the 215 CpG sites with increased methylation in live infected versus control cells; **B**: localization of the 228 CpG sites with decreased methylation in live infected versus control cells. CpG sites that gain methylation (in A) occur predominantly in regions of low CpG density whereas CpG sites that loose methylation (in B) occur predominantly in regions of intermediate CpG density. HC: high CpG density; IC: intermediate CpG density; ICshore: intermediate CpG density shore; LC: low CpG density.

We next tested whether CpGs whose methylation pattern changed specifically in response to *Leishmania* infection shared common genomic characteristics. Of the 215 CpG sites that gained methylation in the live infected versus control cells (Table S3), the majority, 79.5% (171) localize to low CpG density, 16.7% (36) to intermediate CpG density, 3.3% (7) to high CpG density and 0.5% (1) to intermediate CpG density shore (Figure 4A). The enrichment for low density CpG loci was highly statistically significant as determine by hypergeometric test (p-value 4.20e-39). In contrast, in the group of CpG sites that lost methylation, 56.6% (129) localize to intermediate CpG density, 21.9% (50) to high CpG density, 17.1% (39) to low CpG density and 4.4% (10) to low CpG density shore (Figure 4B). Using a hypergeometric test, we found that the enrichment for intermediate density loci was highly statistically significant (p-value 7.15e-128).

### Functional classification of identified genes carrying differentially methylated CpG sites

Next, we tested for functional enrichment among the 315 CpG sites belonging to an annotated gene in our high confidence set of CpGs (Table S3). Using the web-accessible Database for Annotation Visualization and Integrated Discovery (DAVID) v6.7 [22], [23], we identified a number of participating genes of the chemokine signaling pathway, the calcium signaling pathway, the Notch signaling pathway, as well as genes involved in natural killer cell mediated cytotoxicity and others (Table S4). All enriched pathways are listed in Table 3.

**Table 3:** Pathways that have at least four participating genes of the high confidence set.

enriched pathway	numbers of genes detected from the pathway
Calcium signaling pathway	4
Oxidative phosphorylation	4
Huntington's disease	4
Leukocyte transendothelial migration	4
Regulation of actin cytoskeleton	4
Focal adhesion	4
Purine metabolism	4
Cytokine-cytokine receptor interaction	4
Natural killer cell mediated cytotoxicity	4
Notch signaling pathway	5
Adipocytokine signaling pathway	5
chemokine signaling pathway	5
Wnt signaling pathway	6
Tight junction	6
Insulin signaling pathway	6
Endocytosis	7
Pathways in cancer	7

Selected pathways from Table S4. Selection criteria was the condition of having at least four genes within the pathway listed.

### Pyrosequencing confirms DNA methylation array data

**Figure 5:**
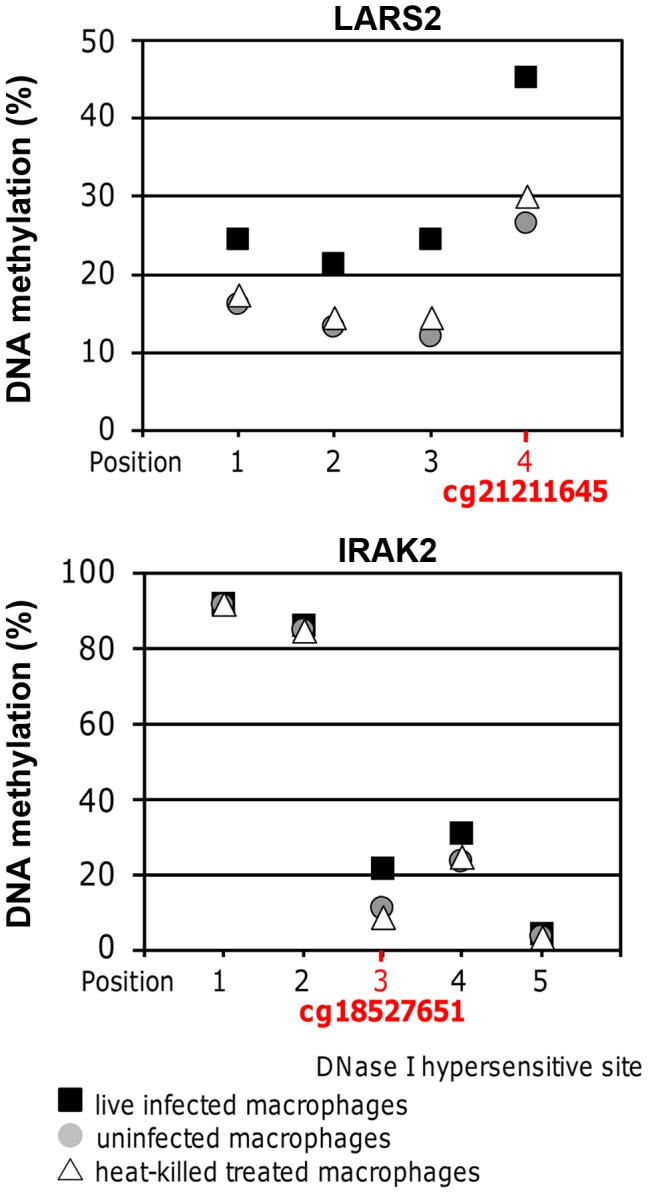
Pyrosequencing confirmed DNA methylation changes of selected high confidence CpG loci. Methylation values at the CpG sites of interest in *LARS2* and *IRAK2* determined by bisulfite PCR-pyrosequencing. The displayed values are the averages of the three biological replicates for live infected macrophages, uninfected macrophages, and heat killed treated macrophages. The methylation at both CpG sites of interest was significantly higher in live infected macrophages compared to the control conditions, showing similar results to the DNA methylation array. A two-tailed t-test was used to determine significance between the experimental conditions (p-value≤0.05).

To validate results of the DNA methylation array, pyrosequencing was performed for the regions containing cg18527651 and cg21211645 in *IRAK2* and *LARS2*, respectively. Cg18527651 and cg21211645 are the top two CpG sites, annotated with a gene name, showing increased methylation when comparing live infected versus heat killed treated cells (Table 2). They are also among the highest differentially methylated CpG sites in live infected versus uninfected (Table 2). The annotated genes, *IRAK2* and *LARS2* play essential roles in immune response of *Leishmania* infected host cells (see discussion) and are thus very interesting candidates to validate. Both, cg18527651 and cg21211645, reside in the 3′UTR of their corresponding gene. The results were consistent with the array, showing a significant increase in DNA methylation in the same three biological replicates of infected cells when compared to either heat killed treated or uninfected cells at both sites of interest (p<0.01; Figure 5). For the *IRAK2* assay, which assessed the methylation at 4 additional CpG sites, one adjacent CpG site showed a similar pattern between the conditions (Figure 5). Furthermore, there was a major difference in DNA methylation values for this amplicon as two of the CpG sites were highly methylated. This difference is likely attributed to a DNase I hypersensitivity site; the 3 CpG sites with decreased methylation values, including cg18527651 reside within it, whereas the two highly methylated CpG sites are located adjacent to it, according to the UCSC genome browser. The hypersensitivity data were taken from ENCODE tracks from UCSC Feb. 2009 (GRCh37/hg19). Since DNase I hypersensitivity sites are generally characterized by open, accessible chromatin, it makes sense that the 3 CpG sites that reside within it are less methylated.

The *LARS2* pyrosequencing assay revealed similar DNA methylation differences between the three experimental conditions at all 3 additional CpG sites assessed in this amplicon, indicating a broad dynamic epigenetic change in this region in host cells upon infection with *L. donovani* (Figure 5).

### Effect on mRNA levels of differentially methylated CpG sites in host macrophages upon infection with *L. donovani*

**Figure 6:**
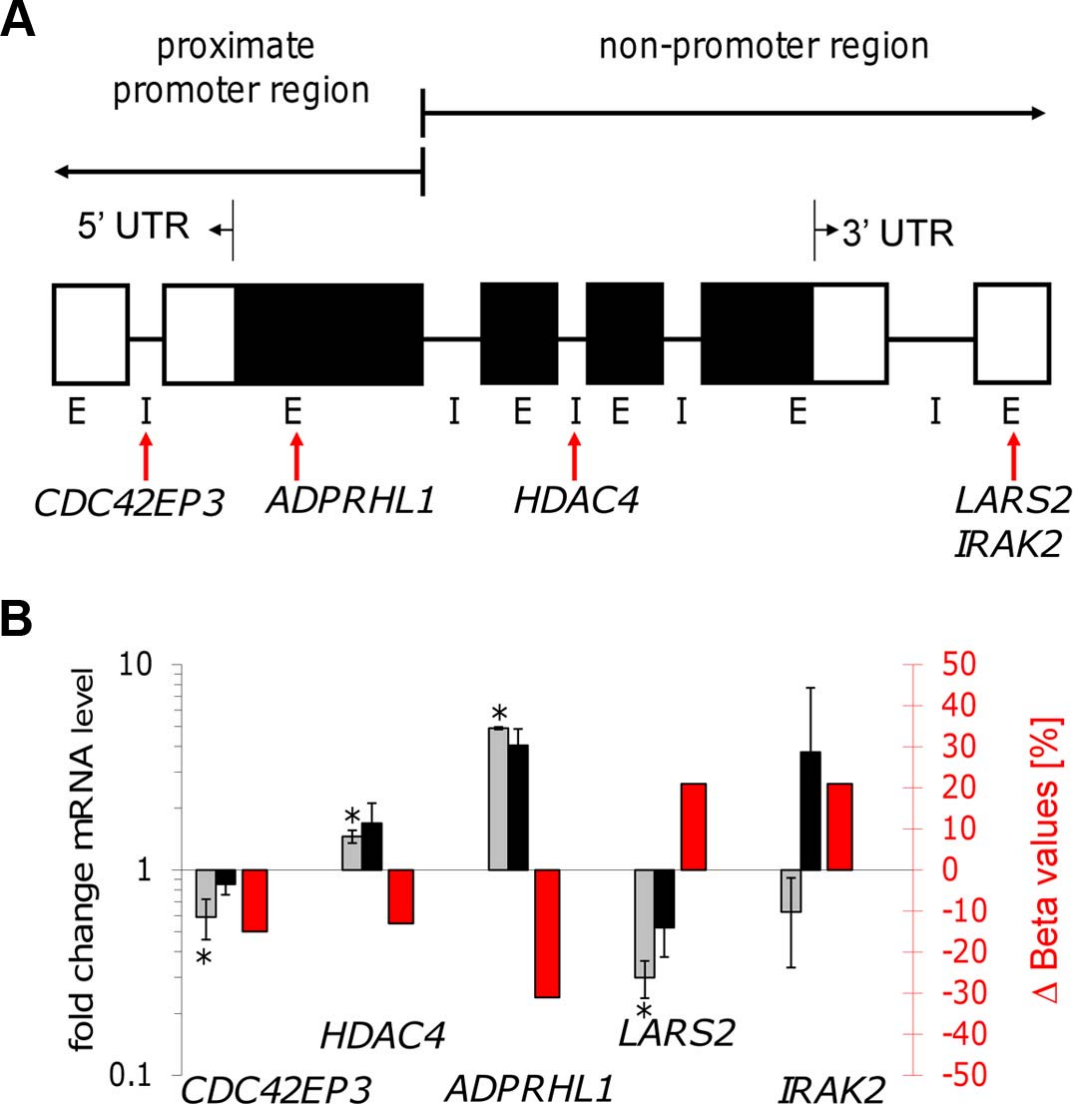
Gene transcription coincided with altered DNA methylation of CpG sites located in promoter and non-promoter regions. **A**: Illustration of the localization of five selected CpG sites. Gene feature annotation in this study is based on Price *et al.* [45]. 5′ UTR: 5′ untranslated region, 3′ UTR: 3′ untranslated region, E: exon, I: intron, white boxes show untranslated exons, black boxes show translated exons, red arrows represent the probe binding sites for the CpG sites of the following five genes: *CDC42EP3*, *ADPRHL1*, *HDAC4*, *LARS2*, *and IRAK2*. **B**: Bar diagram showing differences in DNA methylation (red bars and right y-axis) of five selected CpG sites as well as the fold-change of mRNA level of the corresponding annotated genes comparing live infected versus heat killed treated macrophages (grey/black bars and left y-axis). mRNA level was obtained by real time quantitative RT-PCR analysis. Light grey bars: 72 hr post infection, black bars: 96 hr post infection. * p≤0.005.

To determine whether the changes in CpG methylation observed in macrophages following infection with *L. donovani* resulted in altered gene expression, five genes were selected for further analysis (*CDC42EP3*, *LARS2*, *HDAC4*, *IRAK2*, *ADPRHL1*; listed in Table 2. This group of genes belongs to the high confidence set of CpGs (Table 2) and consists of some that gained and some that lost methylation at their CpG sites. Within the group of genes, the differentially methylated CpG sites are representatives of different localization with respect to the annotated gene, i.e. located in the 5′UTR, the first exon body, the intron body or the exon 3′UTR (see also Figure 6A and Table 4).

**Table 4:** Properties of *LARS2*, *ADPRHL1*, *HDAC4*, *CDC42EP3*, and *IRAK2*.

gene name	gene accession	CpG ID	annotation (a)	methylation In/HK	fold change of transcription (b)	fold change of transcription (c)
LARS2	NM_615340	cg21211645	exon 3′UTR	up (21%)	down (0.3)	0.22
ADPRHL1	NM_138430	cg14339867	1st Exon body	down (−31%)	up (4.91)	NA
HDAC4	NM_006037	cg18845578	intron body	down (−13%)	up (1.45)	1.18
CDC42EP3	NM_006449	cg13286582	intron 5′UTR	down (−15%)	down (0.59)	NA
IRAK2	NM_001570	cg18527651	exon 3′UTR	up (21%)	down (0.62)	0.69

Summary of experimental- and annotation properties of *LARS2*, *ADPRHL1*, *HDAC4*, *CDC42EP3*, and *IRAK2* which are annotated with CpG sites showing increased or decreased methylation in live infected versus heat killed treated macrophages. In: live infected, Hk: heat killed treated. (a) annotation from Price *et al.* [45], (b) determined with qPCR in this study using values for live infected versus heat killed treated macrophages at 72 hr postinfection; (c) determined with DNA microarrays in Dogra *et al.* [3].

Gene expression patterns of all five selected genes were studied by quantifying their mRNA levels in live infected and heat killed treated macrophages using quantitative real time PCR. Since a delayed effect on gene expression might be expected following the alteration of the DNA methylation pattern, mRNA levels were measured at 72 hr and 96 hr post-infection. Figure 6B shows a bar diagram representing the fold difference of mRNA level of each selected gene in comparison to the Δ Beta value of their annotated differentially methylated CpG site. The fold difference of mRNA levels of *LARS2*, *IRAK2* (72 hr time point), *HDAC4*, and *ADPRHL1* were inversely correlated to the Δ Beta value of their differentially methylated CpG site, regardless of the localization of the CpG site. In contrast, the fold difference of mRNA level of *CDC42EP3* and *IRAK2* (96 hr time point) was directly correlated to the methylation pattern of its CpG site: loss of methylation of the CpG site annotated to *CDC42EP3* and located at its 5′UTR, results in lower gene transcription, whereas gain of methylation of cg18527651, located at the exon 3′UTR of *IRAK2* results in elevated mRNA levels after 96 hr infection. Differential gene expression of *CDC42EP3* (p = 0.0014), *HDAC4* (p = 0.0010), *ADPRHL1* (p = 1.5E-05), and *LARS2* (p = 0.0027) was found to be statistically significant for the 72 hr values, whereas *IRAK2* (p = 0.238) was not. For the 96 hr samples, none of the selected genes had a statistically significant change in their gene expression comparing live- versus heat-killed infected THP1 cells.

## Discussion

Epigenetic changes such as DNA methylation and histone modifications play a major role in eukaryotic gene regulation. In this study we demonstrated extensive epigenetic changes in DNA methylation in the host macrophage genome in response to *L. donovani* infection. This was supported by identification of statistically significant differently methylated CpGs between live infected versus heat killed treated macrophages and uninfected macrophages respectively and the absence of differentially methylated CpGs when comparing heat killed to uninfected macrophages. In the high confidence group, there was a slight overrepresentation of CpG loci gaining methylation upon infection. Furthermore, a large fraction of CpGs with altered methylation had substantial overall magnitude of changes of more than 10% (see Table 1). Given that many of the DNA methylation differences currently identified as being associated with disease or environmental exposures are characterized by small absolute changes, often in the range of 5%, this filtering was applied to identify DNA methylation changes that might have a higher likelihood of functional consequences [24], suggesting functional consequences. Consistent with this, targeted mRNA profiling of loci with altered DNA methylation revealed coinciding changes in gene expression.

Interestingly, the genomic features of CpGs that gained DNA methylation upon *L. donovani* infection were strikingly different from those that lost DNA methylation, with the former being enriched for low density loci and the latter being enriched for intermediate density loci respectively.

Many of the differentially methylated CpG sites characterized in this study are annotated to genes whose functions have been previously reported to be modified during a *Leishmania* infection. These include genes coding for proteins involved in signaling pathways such as the JAK/STAT signaling [7], calcium signaling [25], MAPK signaling [26], Notch signaling [27], and mTOR signaling [28], as well as cell adhesion involving integrin beta 1 [29], and changes in host oxidative phosphorylation [30]. We thus propose that *L. donovani* infection induces epigenetic changes in host DNA methylation to enable *L. donovani* survival differentiation and replication within the infected macrophage. Similarly, it was recently reported that *Toxoplasma gondii* induces chromatin remodeling leading to unresponsiveness of its host cells to IFN-γ [18]. In addition, intracellular bacteria and viruses [16], [19], [31], [32] may trigger epigenetic changes in their host cells, an elegant mechanism to alter gene transcription favoring the pathogens infection, replication and survival.

As an integral component of the epigenome, DNA methylation is at the interface between the static genome and changing environments, acting in part through potentially persistent regulation of gene expression. In order to study a possible role of DNA methylation in the modulation of host cell response upon infection with *L. donovani*, we determined host gene expression of five selected genes (*CDC42EP3*, *LARS2*, *HDAC4*, *ADPRHL1*, *IRAK2*) annotated to CpG sites that show a variable methylation pattern between live promastigote infected- and control macrophage DNA samples. We selected five CpG sites with annotated probe binding sites distributed from the 5′UTR, first exon body, intron body and exon 3′UTR (Table 4). In accordance to the differentially CpG methylation pattern in the two condition compared, *CDC42EP3*, *HDAC4*, *ADPRHL1* and *LARS2* showed a statistic significant difference in RNA expression level between live infected- and control samples after 72 hr incubation. The gene expression data after 96 hr infection showed a similar ratio as the 72 hr results, but were not statistically significant. Different gene regulation of the selected genes might thus be a transient event during infection. All genes with statistic significant changes in gene expression in the two conditions tested, except *CDC42EP3*, showed an inverse correlation with DNA methylation (Table 4). It is widely accepted that methylation of CpG sites located in promoter regions specifically (5′UTR, including first exon body) down regulates gene expression while demethylation reverses silencing of genes [14]. This is consistent with our finding that *ADPRHL1* CpG sites were demethylated and corresponding mRNA levels increased, in macrophages infected with live-promastigotes compared to cells exposed to heat killed *Leishmania*. Interestingly, cg14339867, the CpG site annotated to *ADPRHL1*, showed the highest score for demethylation in our comparison (32%, 31% in live infected versus uninfected, live infected versus heat killed treated respectively; see Table 2) and accordingly, the highest ratio of differential RNA-expression among the genes tested in this study (4.91 fold, see Table 4). ADPRHL1 is predicted to be an ADP-ribosylhydrolase like protein that reverses the reaction of ADP-ribosyltransferases, which transfer ADP-ribose from NAD+ to a target protein. Both ADP-ribosylation and de-ADP-ribosylation are posttranslational modifications regulating protein function [33].

Three of the five selected genes (*LARS2*, *IRAK2* and *HDAC4*) are annotated to CpG sites in non-promoter regions (Table 4). Functional interpretation of methylation changes in non-promoter locations of CpG sites such as the gene-body and 3′UTR are more complex and, in contrast to promoter proximate sites, do not follow a linear relationship between methylation and gene expression [14]. However for all three, *LARS2*, *IRAK2* (72 hr value) and *HDAC4*, an inverse correlation was observed between CpG methylation and mRNA expression (Figure 6B, Table 4). The leucyl-tRNA synthetase (encoded by *LARS*) senses intracellular leucine concentration and, in its activated stage, is involved in mTORC1 activation. mTORC1 is a serine/threonine kinase that indirectly regulates gene expression by controlling the translational repressor 4E-BP. The results of the current study demonstrate an increased methylation of the *LARS2*-related CpG site cg21211645 and down regulation of LARS transcription in live infected macrophages compared to control cells suggesting decrease in mTORC1 activity in live infected macrophage cells. Interestingly, we and others have recently demonstrated that upon infection the Leishmania surface zinc metalloprotease GP63 cleaves mTORC1 resulting in inactivation of the mTOR complex1 and activation of the translational repressor 4E-BP1 facilitating *Leishmania* proliferation [28]. Consistent with these results, pharmacological activation of 4E-BPs with rapamycin, results in a dramatic increase in parasite replication whereas infectivity is reduced in 4E-BP1 double knock out mice [28]. *LARS* gene expression was also shown to be downregulated in *L. major* infected macrophages [3] suggesting that this mechanism may be conserved among different *Leishmania* species. Our analysis revealed also CpG site cg11824764, annotated to NM_001163034 (Table S3), with differentially DNA methylation pattern in live infected versus control cells. NM_001163034 is involved in the mTOR pathway (Table S4).

DNA methylation and gene transcription was also inversely correlated for IRAK2 encoding the interleukin-1 receptor-associated kinase 2 that binds to the interleukin-1 receptor and is involved in the upregulation of NF-kappaB leading to gene expression of microbicidal molecules. IRAK2 mRNA level was down regulated 1.5-fold in live infected macrophages compared to heat killed infected macrophages (see Table 4) suggesting a decrease in NF-kappaB levels and activity contributing to an immune silencing mechanism in live infected macrophages. We previously reported down regulation of IRAK2 gene expression in *L. major* infected macrophages [3]. Interestingly, it was demonstrated that *Leishmania* cells escape NF-kappaB induced immune response by preventing the degradation of IkappaB, an inhibitor for NF-kappaB [34]. In addition, elevated levels of ceramide in host cells after *Leishmania* infection was shown to result in the inhibition of NF-kappaB transactivation [35]. Taken together, *Leishmania* cells seem to have developed several independent pathways to inactivate NF-kappaB dependent gene regulation to facilitate onset and progression of successful parasite infection.

We also identified the transcription of *HDAC4* to be up-regulated (1.45-fold) in live infected macrophage samples compared to heat killed infected cells. This up-regulation in *Leishmania* infected macrophages is consistent with DNA microarray studies we previously reported [3]. *HDAC4* encodes a histone deacetylase that is involved in controlling chromatin structure, DNA accessibility and gene expression [36].

*CDC42EP3* mRNA levels were down regulated in host cells upon infection with *L. donovani* (Figure 6B). CDC42EP3 (also called CEP3 [37]) is an effector protein of CDC42, a protein involved in the formation of a protective shell of F-actin around promastigote infected phagosomes [38]. It was suggested that F-actin at higher concentration prevents the phagosomal maturation (a condition favorable to promastigotes until they have differentiated into amastigotes) while in lower concentration might guide lysosomes to phagosomes to enable phagosome-lysosome fusion [39]. In contrast to promastigotes, amastigotes require a phagolysosome environment for survival and successful replication [40]. Thus, downregulation of *CDC42EP3* transcription after a 72 hr and 96 hr infection may be an additional mechanism that *Leishmania* uses to direct host phagosomes to form phagolysosomes to ensure amastigote survival.

Taken together, these data demonstrate significant and likely physiologically relevant epigenetic changes in host cells upon infection with a protozoan pathogen. We propose a new host cell response mechanism upon infection with the parasite *L. donovani*. In this mechanism, invading *Leishmania* parasites trigger methylation changes of specific CpG sites in the host cell genome resulting in an altered gene expression pattern to facilitate *Leishmania* parasite replication and survival. Alternatively the epigenetic changes may be a result of the macrophage innate immune response to *L. donovani* infection. As macrophages are terminally differentiated the epigenetic changes may also be permanent leading to macrophage downregulation of innate immunity. The mechanism of how *L. donovani* may induce epigenetic changes in host cells remains to be determined. The parasite may transfer a factor such as methyltransferase inhibitor or alternative methyltransferases into the macrophage via *Leishmania* exosome secretion or may trigger macrophage factors regulating the methylation machinery.

## Materials and Methods

### Cell culture

THP-1 cells (American Type Culture Collection, Rockville, MD, USA) were cultured in 25 cm^2^ tissue culture flasks containing RPMI-1640 Medium (1x)+2.05 mM L-Glutamine (Thermo Fisher Scientific Inc., Waltham, MA, USA) supplemented with 10% heat inactivated Fetal Bovine Serum (Thermo Fisher Scientific Inc., Waltham, MA, USA), at 37°C in a humidified atmosphere containing 5% carbon dioxide.

*L. donovani* (strain 1S from Sudan, WHO designation MHOM/SD/00/1S-2D) promastigotes were cultured in M199 medium (Thermo Fisher Scientific Inc., Waltham, MA, USA) supplemented with 10% heat inactivated Fetal Bovine Serum (Thermo Fisher Scientific Inc., Waltham, MA, USA), 40 mM HEPES (Mediatech Inc., Manassas, VA, USA), 10 mM hemin (Sigma-Aldrich, St. Luis, USA), 10 U/ml penicillin (Thermo Fisher Scientific Inc., Waltham, MA, USA), and 10 U/ml streptomycin (Thermo Fisher Scientific Inc., Waltham, MA, USA).

### Macrophage infection with *L. donovani*

Viable THP-1 cells, determined with a trypan blue exclusion test, were counted using a hemocytometer. 3×10^5^/ml THP-1 cells were seeded in a tissue culture treated 6-well dish, differentiated for 24 hr using 100 ng/ml PMA (Sigma), washed with complete RPMI medium and allow to rest for 48 hr at 37°C. Differentiated THP-1 cells were infected (MOI 20) with stationary phase live or heat killed (65°C for 45 min) *L. donovani* promastigotes, incubated at 37°C for 24 hrs washed with complete RPMI medium to remove unbound parasites and incubated for an additional 24 hrs (or additional 48 hrs, 72 hrs respectively for the real time PCR experiments), at 37°C before harvesting. To assay for successful phagocytosis of heat-killed *Leishmania* by THP1 cells, stationary phase live *Leishmania* were incubated in 30 µM Vybrant CFDA SE Cell Tracer (Invitrogen) for 45 min at 26°C, washed once with PBS, resuspended in M199 medium and incubated for additional 30 min at 26°C. *Leishmania* were pelleted and resuspended in fresh M199 medium. For the heat-killed samples, *Leishmania* were incubated at 65°C for 45 min. Pre-stained *Leishmania* were then used for infection as explained above. A 24 hr infection time was chosen since heat-killed *Leishmania* get degraded by macrophages at later time points. Confocal images of fluorescently labeled samples were acquired with a Zeiss LSM 780 confocal microscope.

### DNA and RNA extraction of host cells

Total DNA was isolated from three independent infections for each condition (life infected, heat killed treated, uninfected) using All Prep DNA/RNA/Protein Mini Kit (Qiagen Toronto, ON, Canada) following the manufacturers instruction. Total RNA was extracted using All Prep DNA/RNA/Protein Mini Kit (Qiagen Toronto, ON, Canada) following the manufacturers instructions.

### Illumina arrays/statistical analysis

Total DNA was isolated from 48 hr treated (live or heat killed promastigotes) or untreated macrophages as described above and unmethylated cytosines were bisulfite-converted to uracil by using the EZ-DNA Methylation Kit (Zymo Research). After whole genome amplification, DNA was enzymatically fragmented, purified and hybridized to the Illumina Infinium HumanMethylation450 BeadChip arrays (Illumina, San Diego, CA) according to manufacturer protocol. The array contained site-specific probes designed for the methylated- and unmethylated locus of each CpG site covering 99% of the genes from the THP-1 genome. Upon binding of host DNA to their site-specific probes, labeled ddNTPs were incorporated through single-base extension and stained with a fluorescent reagent. Array output was interpreted using the GenomeStudio software from Illumina, after which signal A, signal B, and probe intensities for a total of 485577 CpG sites were exported into R for further processing [41]. First of all, 65 SNP sites residing at rs sites used for subject identification were removed. Then to control for data quality, probes at which one or more samples had undesirable detection p-values (p-value>0.01) or missing measurements were removed, leaving 483329 CpG sites for analysis. Array normalization was performed using color-correction, background subtraction and quantile normalization functions in the Lumi package with default settings [42]. Peak based normalization was then applied to increase data accuracy and reproducibility.

Statistical analysis of the three different experimental conditions was done using Limma to identify significant changes in the methylation pattern.

For this study, M-value was used for all statistical analysis due to its approximate homoscedasticity. M-values have shown to be statistically robust, and it yields better detection and true positive rates for CpG sites that become more or less methylated [43]. Beta value was used for assessing Δ Beta changes and data visualization. To test for differential methylation, we employed the bayesian adjusted t-statistics from the R limma package [44]. First a design matrix was constructed involving the categorical variable that specifies the three different treatments. Then using the design matrix, a linear model was fitted onto the data, after which pair-wise comparisons between the three groups were achieved by constructing a contrast matrix as per specifications in the limma user guide: a) live promastigote infected versus uninfected macrophages; b) live promastigote infected versus heat killed promastigote treated macrophage; c) heat killed promastigote treated versus uninfected macrophages. Multiple testing correction was done using the Benjamini Hochberg (BH) method, and a threshold p-value of 0.05 was used to select for significant differentially-methylated sites for the three comparisons. Change in Beta values was calculated on a probe-wise basis. For probe i, the sample average Beta value was obtained for each of the three treatments, and Δ Beta was calculated according to the following formulas.a) Δ Beta_i,InUn_ = Beta_i,Infected_−Beta_i,Uninfected_ b) Δ Beta_i,InHk_ = Beta_i,Infected_−Beta_i,Heat killed_ c) Δ Beta_i,HkUn_ = Beta_i,Heat killed_−Beta_i,Uninfected_. Lastly, to avoid reporting potential artifacts, more recent annotation of the Human Methylation 450 k array was used to remove from the set of significant CpGs those probes that are known to be polymorphic at the CpG, or have in silico nonspecific binding to the X or Y chromosomes or multiple autosomal loci [45].

Volcano plots for the three comparisons were produced by plotting −log_10_ transformed adjusted p-values on the y-axis, and Δ Beta on the x-axis. The CpG sites passing the significance threshold of 0.05 are marked in red in the upper part of the plot.

### Real-time RT-PCR

Before reverse transcription was performed, RNA samples were tested for the absence of DNA contamination by PCR amplification of the genes to be assayed later with real-time PCR. For cDNA synthesis, 1 µg RNA was reverse transcribed using the QuantiTect Reverse Transcription kit (Qiagen, Toronto, ON, Canada). For quantitative real-time PCR, the QuantiTect SYBR green PCR kit was used (Qiagen, Toronto, ON, Canada) in combination with the QuantiTect Primer Assay (including the synthesized oligonucleotides for *LARS2*, IRAK2, *ADPRHL1*, *HDAC4*, and *CDC42EP3*; Qiagen, Toronto, ON, Canada). Real-time PCR was performed in a StepOne Plus machine (ApliedBiosystems) with cycling conditions following the manufacturer recommendations of the QuantiTect Primer Assay. *ACTB* was used as housekeeping gene to normalize the data and was amplified with the following primer set: forward primer 5′GTTGCGTTACACCCTTTCTT3′ and reverse primer 5′ACCTTCACCGTTCCAGTTT3′ (Integrated DNA Technologies, Coralville, IA, USA). Relative quantification was done using the comparative Ct-method [46]. Gene expression for live- and heat-killed infected THP-1 ΔCt-values were statistically compared by two tailed Student T-test. P-values (p)≤0.005 were considered to be statistically significant after correction for multiple testing (for 10 tests).

### Bisulfite PCR-pyrosequencing

IRAK2 and LARS2 bisulfite PCR-pyrosequencing assays were designed with PyroMark Assay Design 2.0 (Qiagen). The region of interest for IRAK2 was amplified by PCR using the following primers: forward primer 5′AGTATTTTGGGAGTTTGAGGTG3′ and reverse primer 5′BiodTCAAAAATCCATAAAATCTCTTCCTTTCTAA3′(Integrated DNA Technologies, Coralville, IA, USA). Five CpG sites were analyzed by pyrosequencing using sequencing primers 5′GTTTGAGGTGGGAGG3′ and 5′ATGGTATTGTAGAATTGTTAG3′. The region of interest for LARS2 was amplified by PCR using the following primers: forward primer 5′GTTGTGTAGTGAAGTGGAATTAG3′ and reverse primer 5′BiodTCCCTACCTTCCCTCATTAAATATTA3′. Four CpG sites were analyzed by pyrosequencing using sequencing primers 5′GTTTAGGTTGTTGGTTTTAA3′ and 5′AGGTTTTTTAGATGTTGTTT3′. Briefly, a single-strand DNA was prepared from the PCR product with the Pyromar Vacuum Prep Workstation (Qiagen) and the sequencing was performed using the above sequencing primers on a Pyromar Q96 MD pyrosequencer (Qiagen). The quantitative levels of methylation for each CpG dinucleotide were calculated with Pyro Q-CpG software (Qiagen).

## Supporting Information

Figure S1 Individual samples from specific treatments do not cluster next to each other. Unsupervised clustering after normalization of three independent repeats (T1–3) of live infected (In), heat killed (HK), and uninfected (Un).
(TIF)

Table S1 CpG sites with increased or decreased methylation in live infected versus uninfected human macrophages.
(XLS)

Table S2 CpG sites with increased or decreased methylation in live infected versus heat killed treated human macrophages.
(XLS)

Table S3 CpG sites comprising the high confidence set.
(XLS)

Table S4 Pathway analysis of genes belonging to the high confidence set.
(XLSX)
