# Supplementary figures and images for: Leishmania donovani Infection Causes Distinct Epigenetic DNA Methylation Changes in Host Macrophages
Source: PLoS Pathog. 2014 Oct 9;10(10):e1004419. doi: 10.1371/journal.ppat.1004419 (PMC4192605; doi:10.1371/journal.ppat.1004419)

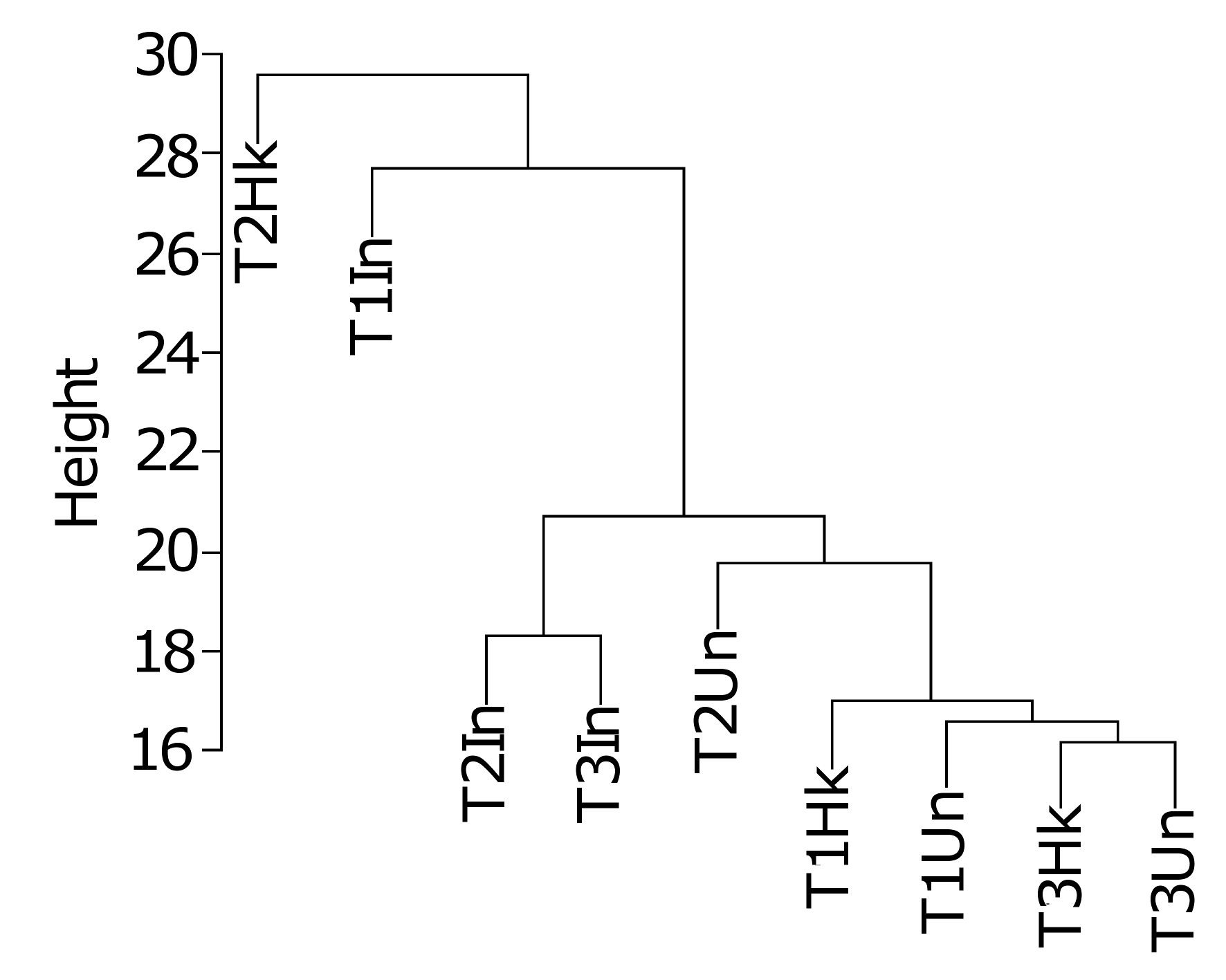

Supplement: Figure S1 — Individual samples from specific treatments do not cluster next to each other. Unsupervised clustering after normalization of three independent repeats (T1–3) of live infected (In), heat killed (HK), and uninfected (Un). (TIF) [file ppat.1004419.s001.tif]
